# Are There Differences in Concentric Isokinetic Strength Perfor-Mance Profiles between International and Non-International Elite Soccer Players?

**DOI:** 10.3390/ijerph18010035

**Published:** 2020-12-23

**Authors:** Robert Śliwowski, Jakub Marynowicz, Monika Grygorowicz, Andrzej Wieczorek, Łukasz Jadczak

**Affiliations:** 1 Department of Team Sports Games, Poznan University of Physical Education, 61-871 Poznan, Poland; marynowicz@awf.poznan.pl (J.M.); wieczorek@awf.poznan.pl (A.W.); lukasz.jadczak@onet.pl (Ł.J.); 2 Department of Physiotherapy, Poznan University of Medical Sciences, 61-701 Poznan, Poland; grygorowicz@ump.edu.pl; 3 Rehasport Clinic FIFA Medical Centre of Excellence, 60-201 Poznan, Poland

**Keywords:** muscular strength profile, quadriceps, hamstring, H/Q ratio, total work

## Abstract

The purpose of this study is to evaluate the differences in concentric isokinetic strength characteristics of the knee extensor and knee flexor musculature between international (IL) and non-international level (N-IL) soccer players. The second aim is to establish strength symmetry status in knee muscles for dominant (DL) and non-dominant (NDL) legs for both within and between groups. 100 male top elite soccer players (IL: *n* = 36, age = 27.5 ± 3.4 years and N-IL: *n* = 64, age = 27.7 ± 6.4 years) underwent concentric isokinetic strength tests, using a Biodex System 3 dynamometer. Results indicate that statistically significant differences between groups were noted for peak torque of hamstrings (PT-H), hamstrings/quadriceps (H/Q) ratio, and total work of hamstrings (TW-H), where mean values for the IL were similarly higher than for the N-IL group (*p* = 0.006, *p* < 0.001, and *p* = 0.012, respectively). Our results also showed statistically significant differences for peak torque of quadriceps (PT-Q), PT-H, total work of quadriceps (TW-Q) and TW-H between legs, where mean values noted for the DL were higher than for the NDL for both groups (*p* = 0.021, *p* < 0.001, *p* = 0.006, and *p* = 0.004, respectively). Additional results show that IL players presented more symmetrical strength between legs than N-IL. The results of this study indicate that that the greatest differences in isokinetic strength performance across players at different soccer levels relate to the hamstring muscle. As a result, systematic strength training of these muscle groups is strongly recommended.

## 1. Introduction

Isokinetic testing is one of the most commonly used methods of assessment for a wide range of strength evaluations. Traditionally, the focus of isokinetic research has been the measurement of concentric and eccentric absolute peak torque (PT) of quadriceps and hamstrings. Away from PT, the most commonly described variables in isokinetic tests for soccer players’ knee muscles are: hamstring/quadriceps ratio, total work, average power, and fatigue index. The evaluation of muscular isokinetic strength allows for the determination of soccer players’ muscular performance profiles and is therefore relevant for both maximization of physical performance and injury prevention. 

Previous research has identified the isokinetic strength of knee extensor and flexor muscles as differentiating factors between soccer players of playing levels [1,2,3,4,5,6,7] and positional differences [8,9,10,11]. Some prior research indicates that soccer playing leads to a significant increase in strength in the muscles surrounding the knee [12]. Other studies have found hamstring and quadriceps strength increases, albeit to different degrees. For example, Lehance et al. [4] reported that concentric PT of the extensors and flexors increases with the age and sporting level of soccer players. The earlier study of Oberg et al. [5] also indicated that concentric isokinetic PT of the quadriceps and hamstrings increased in line with level of play in Swedish soccer divisions. The same study found that international soccer players were stronger than non-internationals. Conversely, Cotte and Chatard [3], found that international soccer players were no stronger than national players. Another study indicated that the more years of training professional soccer players had, the higher the PT of their knee extensors and flexors [13]. It should be noted that, based on comparisons of PT values between young and adult players, it has been suggested that soccer playing may be conducive to greater development of the hamstrings [14]. This phenomenon is also confirmed by the study of Cometti et al. [1] which indicates that the only parameter that differs between elite and amateur players is concentric knee flexor strength. Moreover, that study also found that hamstrings were stronger in professional players than in amateurs. This, in turn, may indicate that it is the level of hamstring muscle strength that differentiates soccer players at various levels. 

The significance of differences in muscle strength between legs continues to be a hotly debated question in the existing research. Soccer players rarely use both legs with equal emphasis, often favouring the use of their dominant limb when performing game-specific activities [15]. This can result a stronger dominant leg (DL) compared to the non-dominant leg (NDL) [16,17,18]. A number of studies [9,19,20] have indicated no significant difference between the two extremities, while other studies [7,17] have found significant superiority of DL over NDL with regard to concentric strength. In contrast, other studies [16,21,22] have showed that either knee flexors of the dominant side to be significantly stronger than those of the non-dominant side or vice versa (i.e. NDL knee flexors can be significantly stronger than DL knee flexors) [23]. Additionally, there may be many combinations of muscular asymmetries within the type of muscular contraction (concentric vs. eccentric), leg (D vs. NDL) and a given muscle group (flexors vs. extensors). It may be expected that with a constant increase in research on this specific topic (i.e., inter- and between-limb asymmetries) and with the change in approach to preventative training, the differences in muscle strength between legs will gradually decline in the coming years. Thus, as can be expected, explanations for this discrepancy is still a subject of debate.

Despite the growing popularity of between limb asymmetry topic at different levels, there is a lack of specific research regarding the differences in isokinetic strength performance between soccer players at highest competition level. There is a similar knowledge deficit surrounding about differences in the concentric isokinetic strength characteristics of the knee extensor and knee flexor musculature between international and non-international soccer players. The advancement of such knowledge may have important implications for soccer training programs.

Therefore, the aim of the current study is to evaluate the differences in concentric isokinetic strength characteristics of the knee extensor and knee flexor musculature between international and non-international soccer players. The second aim is to establish strength symmetry status in knee muscles for dominant and non-dominant limbs for both within- and between-limb groups. To our knowledge, this information will be first of its kind that uses soccer as the sporting context. 

## 2. Material and Methods 

### 2.1. Participants and Data Collection 

The cohort for this study included 100 male soccer players, 36 who played at an international level (IL) and 64 at a professional (non-international) elite level (N-IL). The IL group (age: 27.5 ± 3.4 years; height: 181.8 ± 5.1 cm; body mass: 77.3 ± 5.5 kg; training experience: 17.1 years) included international players from the Polish Ekstraklasa (the top competition level in Poland), who had played a minimum of 10 games for national teams of their respective countries (mainly Poland and countries of Southern, Eastern and Northern Europe). The N-IL group (age: 27.7 ± 6.4 years; height: 182.3 ± 2.7 cm; body mass: 79.4 ± 8.9 kg; training experience: 16.8 years) consisted of Polish Ekstraklasa players, without experience playing for a national team. 

All participants from the IL and N-IL groups had at least three years of professional soccer playing experience, with regular training (as expounded in their contracts). The study was carried out from 2010 to 2019. Every measurement was performed twice per year: at the beginning of the pre-season period (from January to February), and from June to July. The official start of the season was in August every year. During every season, each participant completed two functional movement screen tests, as well as isokinetic strength tests for hamstring quadriceps, and knee, proprioception tests, and ground reaction force analysis, which were part of their routine biomechanical evaluation. As an annex to their professional contracts, players were given information about the experimental risks and provided written consent for their data to be collected and used. In cases where players below the age of 18, parents or guardians were informed about the risks and their written, informed consent was obtained before commencement of the study. The study was conducted in accordance with the Declaration of Helsinki and the research protocol was approved by the local research ethics committee (the Bioethical Committee at the Poznań University of Medical Sciences).

### 2.2. Test Procedures 

The tests in this study were carried out at the Rehasport Clinic, a FIFA Medical Centre of Excellence in Poznań, Poland. All measurements were performed by the same team of examiners, who had completed additional specialized biomechanical evaluation courses. In addition, each examiner had at least 3 years of isokinetic joint testing experience, including knee joints. The Biodex System 3 (Biodex Corp, Shirley, NY, USA) dynamometer was used to measure isokinetic knee muscle strength (ascertained by PT and muscle endurance (as measured by total work [TW])). 

With regard to the participants’ alignment axis of dynamometer rotation, position, gravity correction, and stabilization, all procedures followed guidelines previously described in existing literature [19,24,25]. Before the isokinetic assessment, each player performed a 10–15 min warm-up, which entailed pedaling on a Monark 828E Ergomedic stationary cycle ergometer (Monark, Vansbro, Sweden) at a moderate pace (50–100 watt) and dynamic stretches for the major lower-limb muscle groups [19,23]. To assess the concentric isokinetic torque of the quadriceps and hamstrings, continuous, bidirectional knee extension-flexion movements were completed at an angular velocity of 60°·s^−1^ and 240°·s^−1^ through a knee range of motion of 0° (flexed) to 90° (full extension). The above testing speeds of 60°·s^−1^ and 240°·s^−1^ have been extensively implemented in other studies looking at muscle strength in soccer players [4,12,20]. The participants performed three trials at submaximal efforts, with a load gradually increasing to 50%, 75%, and approximately 100% of maximum capability, followed by one set of three repetitions at the maximal concentric contraction at angular velocities of 60°·s^−1^, and 30 repetitions at angular velocities of 240°·s^−1^. The same procedure was then repeated for the remaining leg. After the third submaximal trial, the participants were given a 30-s rest, and a further, one-minute break between two angular velocities, followed by a three-minute break. During this break, machine settings was altered for the opposite leg). Participants were also asked to complete the movement in full range of motion. The order of testing was randomized for the dominant (DL) and non-dominant (NDL) legs [16,19,25]. dominance player’s dominant limb was determined on the basis of their preference when kicking a ball [18,19]. Only windowed data was used in order to limit the analysis to constant velocity periods. In the statistical analysis, the following data were analyzed: relative PTs (normalized by the body weight and expressed in Nm·kg^−1^) for flexors (PT-Q) and extensors (PT-H) in both legs, the unilateral ratio of muscle torque for both the dominant and non-dominant extremities (HDL/QDL and HNDL/QNDL, respectively) at an angular velocity of 60º·s^−1^ and, similarly, the relative TWs (J·kg^−1^) for extensors (TW-Q) and flexors (TW-H) for both legs at an angular velocity of 240°·s^−1^.

All tests were performed before 1 pm to exclude inter-day variability and were performed in the same order for each participant. Players undergoing the evaluation were exempted from intensive training for 48 h prior to testing. Before commencement of the testing, participants were asked to complete a questionnaire to determine whether they had any musculoskeletal pain, discomfort, or known injury in a lower extremity. Participants reporting either a major or moderate lower leg injury or any injury to the knee or thigh were excluded from further analysis. None of the participants had prior significant knee injuries, none had a history of anterior cruciate ligament (ACL) repairs or rehabilitation, and none had a history of a leg fracture or surgery during the year preceding each evaluation. 

### 2.3. Statistical Analysis

All statistical analyses were performed using Statistica Version 13.0 (StatSoft Polska Sp. z o.o. 2020, Krakow, Poland). Descriptive data are presented as means and standard-deviation, whereas percentage difference in the variables between dominant and non-dominant leg is expressed as an absolute value of mean with 95% confidence limits. The normality of each variable was initially tested with the Kolmogorov-Smirnov test, the coefficients of asymmetry and kurtosis were also ascertained. All the variables presented a normal distribution. Five separate two-way mixed analyses of variance (ANOVA) were used to evaluate the effects of groups (between subject factor, levels: international vs. non-international level) and legs (within subject factor levels: dominant vs. non-dominant), and their interactions on dependent neuromuscular variables (PT-Q, PT-H, H/Q ratio, TW-Q and TW-H). F-ratios (F), degrees of freedom subscripted, *P* value (*P*), and effects sizes (partial η^2^) were reported for each ANOVA. A paired *t*-test was also applied to examine differences between the scores of the dominant and non-dominant legs for the all neuromuscular variables in both cohorts independently. T statistics (*t*), degrees of freedom subscripted, *P* value (*P*), and Cohen’s *d* (*d*) effect sizes were reported for each *t*-test. Cohen’s *d* was determined as a measure of effect size for between leg comparisons. Cohen’s *d* lower than 0.2 was considered irrelevant, between 0.2 and 0.49 was small, between 0.50 and 0.8 was considered medium, and greater more than 0.8 was considered high [26]. The level of statistical significance was at 0.05 for all statistical procedures.

## 3. Results

**Figure 1:**
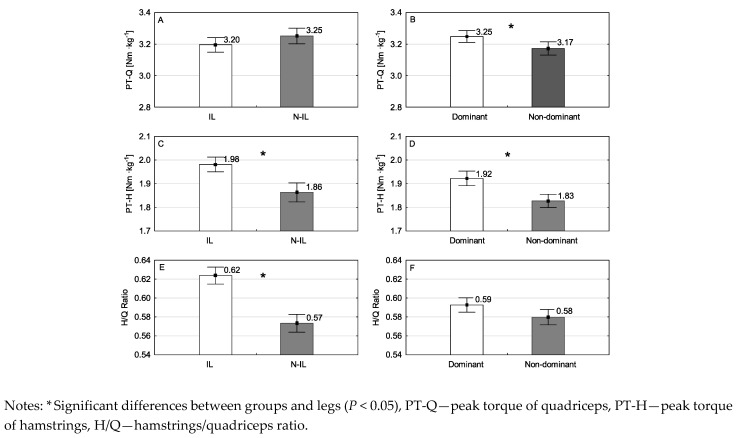
Mean values and standard error for PT of quadriceps and hamstrings by: (**A**,**C**) soccer player groups (international, non-international); (**B**,**D**) legs (dominant, non-dominant), and H/Q ratios by: (**E**) soccer player groups (international, non-international); (**F**) legs (dominant, non-dominant).

**Figure 2:**
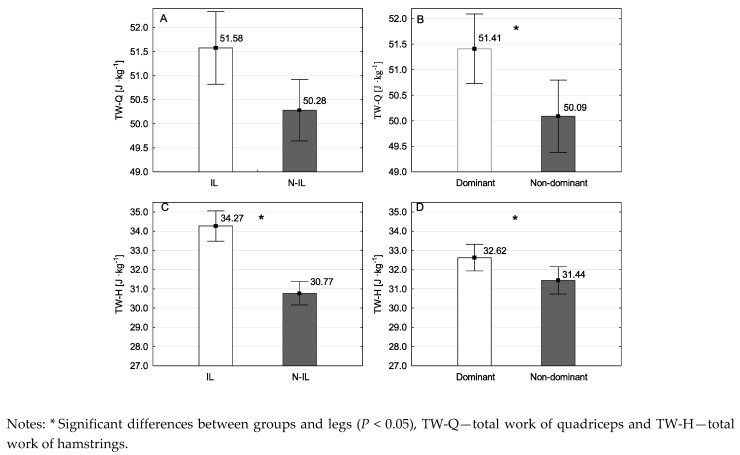
Mean values and standard error for TW of quadriceps and hamstrings (**A**,**C**) across soccer player groups (international, non-international); (**B**,**D**) legs (dominant, non-dominant).

There were no significant differences in height or weight across the studied participants. Figure 1 and Figure 2 presents the mean values of PT-Q, PT-H, H/Q, TW-Q and TW-H for groups and legs. For PT-Q there was no interaction (F_1,98_ = 0.210, *P* = 0.648, partial η^2^ = 0.002) and no main effect for groups (F_1,98_ = 0.004, *P* = 0.949, partial η^2^ < 0.001; Figure 1A). However, there was a main effect for legs (F_1,98_ = 5.516, *P* = 0.021, partial η^2^ = 0.053; Figure 1B), where the mean values for DL values were greater than NDL. For PT-H there was no interaction (F_1,98_ = 0.036, *P* = 0.850, partial η^2^ < 0.001). However, there was a main effect for groups (F_1,98_ = 7.903, *P* = 0.006, partial η^2^ = 0.075; Figure 1C), where the mean values for IL were greater than N-IL, and for legs (F_1,98_ = 23.503, *P* < 0.001, partial η^2^ = 0.193; Figure 1D), where the mean values for DL were greater than NDL. For H/Q ratio there was no interaction (F_1,98_ = 0.095, *P* = 0.758, partial η^2^ < 0.001) and no main effect for legs (F_1,98_ = 3.871, *P* = 0.052, partial η^2^ = 0.038; Figure 1F). However, there was a main effect for groups (F_1,98_ = 13.574, *P* < 0.001, partial η^2^ = 0.122; Figure 1E), where the mean values for IL were greater than N-IL. For TW-Q there was no interaction (F_1,98_ = 0.830, *P* = 0.364, partial η^2^ = 0.008) and no main effect for groups (F_1,98_ = 0.878, *P* = 0.351, partial η^2^ = 0.009; Figure 2A). However, there was a main effect for legs (F_1,98_ = 7.871, *P* = 0.006, partial η^2^ = 0.074; Figure 2B), where the mean values for DL were greater than NDL. For TW-H there was no interaction (F_1,98_ < 0.001, *P* = 0.992, partial η^2^ < 0.001; Figure 2C). However, there was a main effect for groups (F_1,98_ = 6.522, *P* = 0.012, partial η^2^ = 0.062; Figure 2C), where the mean values for IL were greater than N-IL, and for legs (F_1,98_ = 8.520, *P* = 0.004, partial η^2^ = 0.079; Figure 2D), where the mean values for DL were greater than NDL.

Table 1 presents the mean values of neuromuscular characteristics for both groups. Statistically significant differences were only reported in the international group for PT-H and TW-H (t_35_ = 3.756, *P* < 0.001, *d* = 0.42, and t_35_ = 2.321, *P* = 0.026, *d* = 0.18, respectively), where the mean DL values were greater than NDL. In the case of the non-international group, statistical significance was noted for PT-Q, PT-H, TW-Q and TW-H (t_63_ = 2.362, *P* = 0.021, *d* = 0.19; t_63_ = 3.597, *P* < 0.001, *d* = 0.29; t_63_ = 3.154, *P* = 0.003, *d* = 0.22 and t_63_ = 2.204, *P* = 0.031, *d* = 0.17, respectively), where mean values for DL were higher than for NDL.

## 4. Discussion 

The primary findings of the present study reveal that isokinetic flexors characteristics (i.e. PT-H and TW-H), and H/Q ratio were significantly greater for international soccer players compared to non-internationals. Notably, no statistically significant differences were noted for PT-Q and TW-Q between the compared groups. These findings suggest that specific strength adaptations in muscular architecture among knee joints in international players are mainly manifested in the flexor muscle group, which could directly translate into higher values of H/Q ratio in this group.

These results are in accordance with the findings of Cometti et al. [1], who found that experienced professional soccer players had stronger knee flexors and higher conventional H/Q ratios than amateur players, thus highlighting the effect of level of play. Similar to our findings, above, they found a comparable level of quadriceps strength across the groups. A comparable phenomenon was described in a recent study by Herdy et al. [27], where professional adult soccer players demonstrated significant differences in hamstring PT in both legs, compared to U-17 players. As in our study, statistically significant differences were also noted among the same groups in the case of a conventional H/Q ratio; however, these differences related only to DL. A number of cohort studies [5,28] have suggested that players from higher leagues produce higher PT values during concentric extension and flexion actions, compared to players in lower divisions or leagues [28]. They attributed the greater strength of high-level soccer players to longer off-season periods and also to a greater number of specific strength training sessions. These relations, however, were not confirmed in another study, which found no significant differences in the PT of the quadriceps and hamstrings, nor the H/Q ratio, across four divisions of soccer players [6], nor between international and national soccer players [3]. In relation to the total work variable, the only study in this area known to us [2] found statistically significant differences with regard to both quadriceps and hamstrings—between professional and U-17 soccer players for all the studied angular velocities. Unfortunately, wider comparisons of TW with other elite players is not available in the current literature.

These observations indicate that many years of changes in knee muscular strength and endurance in soccer players can have various results. Soccer training is likely to have significant potential for both quadriceps and hamstring muscle group strengthening, but a clear effect in our study seems relatively higher for the hamstring muscle in international players. It seems that experienced professional players, performing at a high level exhibit a better strength balance around the knee joint and stronger knee flexors than lower level players. It is generally accepted that the quadriceps muscle group plays an important role in kicking, passing and jumping—primarily in the concentric mode [8,10,11,12]. By contrast, hamstrings are largely used eccentrically to control, decelerate, and stabilize the knee, although it should be noted that they are also used concentrically in actions such as tackling, high velocity running, and turning [8,10,11,24]. Many studies indicate that a player’s high-intensity activity during a match is influenced by training level [29] and the level of competition [30]. More precisely, Thorpe and Sunderland [31] revealed that semi-professional players have been found to cover lower total distances and distances at high velocities. At the same time, modern soccer is becoming faster and more dynamic and as such, high-intensity running distances and high-intensity actions during a game have increased exponentially in recent years [32]. Altogether, these differences can generate different musculature adaptations between players representing various sports levels, especially with regard to flexor muscles. As a result, the significantly higher PT-H and TW-H values observed in the international soccer players in our study are likely a result of increased game exposure. Compared to non-international players, the higher number of matches played per season (more than 70 matches played per season) among international players increased the number of high-intensity efforts performed, such as high-speed running or sprints [33]. There is a substantial body of evidence showing correlatory relationships between, on the one hand, the eccentric [34,35,36] and concentric [4,35] strength of the hamstring, with, on the other hand, speed performance. Morin et al., [37] highlighted the crucial role of hamstring muscles in producing propulsive force during acceleration performance. Furthermore, hamstrings play an important role in producing the required joint moments when running at high velocities. Moreover, Exell et al. [38] found that hamstrings are instrumental in accelerating the center of mass when performing fast consecutive hips-extensions and are also important for increasing step frequency and speed when performing fast knee-flexions. Such findings indicate the need for further research in this area, especially with regard to concentric contractions for flexor muscles, as has been done in the present study.

Compared to the non-international group soccer players, the higher H/Q ratios shown in the international group in our study can thus be explained by their stronger hamstrings. It should be noted though that PT-Q strength did not show statistically significant differences between the compared groups of soccer players (Table 1). It should also be born in mind that the H/Q ratio is not only a significant indicator in the prevention of injuries [12,19,24], but also reflects the relatively important relationship between the strength generated between knee flexors and extensors during specific functional efforts and the type of movement activities performed on the pitch. The higher values of H/Q ratio in the international group may indicate better strength balance around the knee joint than in the non-international group. Indeed, research suggests that strength balance around the knee joint is better in experienced professionals playing at a high level than it is in lower level players (see also [1,13]). Moreover, it is widely accepted that high hamstring strength can be used as a preventative neuromuscular strategy against over-fatiguing and subsequent injury [39]. 

Although modern training programs focus on the two-legged preparation of players, some research [13,18] has suggested that soccer players seldom use both legs with equal emphasis. In general, more experienced players may show a greater tendency for asymmetry due to a natural preference for their left or right foot when performing particular movement patterns [13]. These findings are consistent with our own results, but only if we take into consideration the non-international group, which found statistically significant differences in PT-Q, PT-H, TW-Q and TW-H between players’ legs (Table 1), where mean values noted for the DL were higher than for the NDL. Greater symmetry between legs was reported in the international group, and, interestingly the symmetry related mainly to the quadriceps, and was not apparent for hamstrings. We hypothesize that this pattern is a result of a greater manifestation of two-leggedness in the play of the international group, however it can also be a compensatory effect of other musculature such as hip flexors/extensors or ankle. An analysis of patterns of shooting on goal during top-level competition revealed that most successful players shot for goal using both left and right lower legs [40]. More recent research has emphasized the importance of using both feet equally in soccer [15]. It is worth mentioning that larger lower limb strength asymmetries may negatively affect jumping ability and power output and hence limit athletes’ potential [41]. In addition, we assume that magnitude of symmetry can be associated with playing position, and therefore further research should focus on position-specific asymmetry. 

Differences between DL and the NDL for soccer players are a controversial subject, as some studies demonstrate various ranges of musculoskeletal asymmetries in the knee extensor and flexor strength. The latest meta-analysis by DeLang et al. [42] indicates that soccer players across all ages and levels demonstrate between-limb symmetry in knee extensor and flexor peak torque, as well as H/Q ratio, regardless of concentric or eccentric measurements. These findings are confirmed by a number of other studies which have found no statistically significant differences in the level of PT-Q, PT-H or conventional H/Q ratios between extremities [7,10,15,20,25]. The study by Fousekis et al. [17] provides additional data indicating that fluctuating asymmetries between DL and NDL among semi-professional soccer players from the Greek third division relate mainly to eccentric work and no significant tendency toward asymmetry was noted when concentric measurements were applied. Unlike our findings, in Fousekis et al.’s view [17], this phenomenon demonstrates that asymmetric strength adaptations taking place in the lower extremities of professional soccer players are mainly eccentric in nature. Elsewhere, similar to our study, the superiority of the concentric isokinetic strength in DL was observed in at 60° s^−1^ angular velocity [7] and number of other studies have found that knee flexors [16,21,22] and extensors [18] of the dominant leg were significantly stronger than those of the non-dominant one. Such data, together with the results of our study, strongly support the theory that various degrees and modes of functional asymmetry develop as a result of long-term participation in soccer [17]. 

As with all such research, this study has some limitations, which should be elucidated. The main limitation of the current study is that measurement procedures were based exclusively on the concentric contractions mode. Additionally establishing eccentric PT-H could not only reveal some interesting relationships between concentric and eccentric PT-H, but also enable the calculation of dynamic H/Q ratios and would thus contribute to a complete report of the muscle profile. 

## 5. Conclusions 

The principal finding of this study is that the greatest differences in isokinetic strength performance across players at different soccer levels relate to the hamstring muscle. It is noteworthy that this phenomenon relates to isokinetic indicators of both strength and strength endurance. However, no significant differences in these areas was observed in the quadriceps muscle group. It appears that the intergroup heterogeneity in terms of hamstring muscle strength may result from the difference in training adaptations and the level of competition of the analysed players. It thus seems logical to infer that knee flexor strength is extremely important in soccer players for repeated high-intensity bouts of activity and for joint stabilisation during various tasks; systematic strength training of these muscle groups is therefore highly recommended. We also found statistically significant intergroup differences in H/Q ratios. A higher level of this indicator, along with a greater reciprocal ratio, and greater hamstring muscle strength may indicate better performance and strength balance around the knee joint in the international players than non-international, as well as a greater potential for muscular joint stabilisation. Other results show that international players presented more between-leg symmetrical strength than non-international players. These findings indicate that specific kinetic patterns found in international soccer could be of more balanced and symmetrical nature. Finally, we believe that further research of this type should be complemented with measurement of the eccentric contractions mode, particularly with reference to hamstring muscles.

## Figures and Tables

**Table 1:** Characteristics of the PT of quadriceps and hamstrings, H/Q ratios at 60°·s^−1^ and TW of quadriceps and hamstrings at 240°·s^−1^ angular velocity across soccer player groups.

Variables	International Level (n = 36)			Non-International Level (n = 64)		
	DL	NDL	Δ% 95% CL	DL	NDL	Δ% 95% CL
	Mean ± SD			Mean ± SD		
PT-Q [Nm·kg^−1^]	3.24 ± 0.35	3.18 ± 0.41	7.95 (6.0 − 9.9)	3.25 ± 0.39 *	3.17 ± 0.44	6.95 (5.4 − 8.5)
PT-H [Nm·kg^−1^]	2.03 ± 0.26 *	1.93 ± 0.22	6.67 (4.9 − 8.5)	1.86 ± 0.32 *	1.77 ± 0.30	9.82 (8.1 − 11.6)
H/Q Ratio	0.63 ± 0.06	0.61 ± 0.08	8.55 (6.5 − 10.6)	0.57 ± 0.08	0.56 ± 0.08	9.13 (7.4 − 10.9)
TW-Q [J·kg^−1^]	51.99 ± 6.46	51.17 ± 6.42	7.11 (5.4 − 8.8)	51.08 ± 7.03 *	49.48 ± 7.41	6.75 (5.2 − 8.3)
TW-H [J·kg^−1^]	34.86 ± 6.31*	33.67 ± 7.09	7.37 (5.3 − 9.4)	31.36 ± 6.94 *	30.18 ± 6.89	11.87 (9.9 − 13.9)

Notes: * Significant differences between legs (*P* < 0.05), PT-Q—peak torque of quadriceps, PT-H—peak torque of hamstrings, H/Q—hamstrings/quadriceps ratio, TW-Q—total work of quadriceps and TW-H—total work of hamstrings.

## Data Availability

The data are not publicly available due to Rehasport restrictions. Requests for the data and information for the Rehasport Clinic Institutional Data Access can be sent to Joanna Wiese, joanna.wiese@rehasport.pl.
